# Factors influencing unmet need for family planning among Ghanaian married/union women: a multinomial mixed effects logistic regression modelling approach

**DOI:** 10.1186/s13690-019-0340-6

**Published:** 2019-03-12

**Authors:** Chris Guure, Ernest Tei Maya, Samuel Dery, Baaba da-Costa Vrom, Refah M. Alotaibi, Hoda Ragab Rezk, Alfred Yawson

**Affiliations:** 1 Department of Biostatistics, School of Public Health, University of Ghana, Legon, Accra Ghana; 2 Department of Population, Family and Reproductive Health, School of Public Health, University of Ghana, Legon, Accra Ghana; 3 Department of Mathematical Sciences, Faculty of Science, Princess Nourah bint Abdulrahman University, Riyadh, Saudi Arabia; 4 Princess Nourah bint Abdulrahman University, Riyadh, Saudi Arabia

**Keywords:** Unmet need, Family planning, Estimating unobserved effects, Multilevel modelling

## Abstract

**Background:**

Unmet need for family planning is high (30%) in Ghana. Reducing unmet need for family planning will reduce the high levels of unintended pregnancies, unsafe abortions, maternal and neonatal morbidity and mortality. The purpose of this study was to examine factors that are associated with unmet need for family planning to help scale up the uptake of family planning services in Ghana.

**Methods:**

This cross sectional descriptive and inferential study involved secondary data analysis of women in the reproductive age (15–49 years) from the Ghana Demographic and Health Survey 2014 data. The outcome variable was unmet need for family planning which was categorized into three as no unmet need, unmet need for limiting and unmet need for spacing. Chi-squared test statistic and bivariate multilevel multinomial mixed effects logistic regression model were used to determine significant variables which were included for the multivariable multilevel multinomial mixed effects logistic regression model. All significant variables (*p* < 0.05) based on the bivariate analysis were included in the multinomial mixed effects logistic regression model via model building approach.

**Results:**

Women who fear contraceptive side effects were about 2.94 (95% CI, 2.28, 3.80) and 2.58 (95% CI, 2.05, 3.24) times more likely to have an unmet need for limiting and spacing respectively compared to those who do not fear side effects. Respondents’ age was a very significant predictor of unmet need for family planning. There was very high predictive probability among 45–49 year group (0.86) compared to the 15–19 year group (0.02) for limiting. The marginal predictive probability for spacing changed significantly from 0.74 to 0.04 as age changed from 15 to 19 to 45–49 years. Infrequent sexual intercourse, opposition from partners, socio-economic (wealth index, respondents educational level, respondents and partner’s occupation) and cultural (religion and ethnicity) were all significant determinants of both unmet need for limiting and spacing.

**Conclusions:**

This study reveals that fear of side effect, infrequent sex, age, ethnicity, partner’s education and region were the most highly significant predictors of both limiting and spacing. These factors must be considered in trying to meet the unmet need for family planning.

**Electronic supplementary material:**

The online version of this article (10.1186/s13690-019-0340-6) contains supplementary material, which is available to authorized users.

## Background

Beyond the health benefits that accrue to women, children and men from family planning, it is a catalyst for environmental sustainability, [1] and economic growth of countries [2]. Thus, Ghana’s strive to improve its economic fortunes and health of the populace will be difficult if efforts are not made to reduce its high unmet need for family planning.

Unmet need for family planning is essentially the percentage of married/union women of reproductive age who are not using any method of family planning but who would like to postpone the next pregnancy (unmet need for spacing) or do not want to have any more children (unmet need for limiting) [3]. The concept of unmet need defines the gap between women’s reproductive intentions and their contraceptive behaviour. Unintended pregnancies have serious consequences for the health and well-being of women and their families, particularly in developing countries where maternal mortality is high and induced abortions are often unsafe. More than 358,000 women die of pregnancy-related causes every year, according to a report from the World Health Organization [4]. Couples who use contraception have the ability to control the number and spacing of their children thus preventing unintended pregnancies, abortions and deaths related to pregnancy and childbirth.

The recent Ghana Demographic and Health Survey 2014, estimated that 30 % of currently married women have an unmet need for family planning services, with 17% having an unmet need for spacing and 13% having an unmet need for limiting. Knowledge of contraceptives is universal in the developed world and almost universal in the developing world [5]. Globally, there is a high saturation of knowledge on contraceptive methods, with knowledge of at least one contraceptive method in sub-Saharan Africa being approximately 85%, [6].

The 2014 Ghana Demographic and Health Survey found that 99% of women and men knew of at least one contraceptive method [7]. The survey also showed that modern contraceptive methods were more known than traditional ones among women, with the male condom (96%), injectable (92%), and pills (91%) being the most commonly known methods. However, there is considerable variability in this knowledge across different population demographics such as, age, occupation, religion and ethnicity [8]. Knowledge however does not directly translate to use.

The United Nations [9] report on world contraceptive patterns shows that 63% of women of reproductive age who are married or in a union use a contraceptive method. Globally, female sterilization is the most common method of contraception, used by 19% of married/union women of reproductive age (15–49 years) group. The IUD, used by 14% of women of reproductive age who are married or in a union, is the second most widely used contraceptive method in the world, followed by the pill.

Ghana is a signatory to the Family Planning 2020 (FP2020) and has committed to increasing modern contraceptive use among married/in union women from 22% in 2012 to 30% in 2020, (Government of Ghana (GOG), 2016). In Ghana, the prevalence of modern contraceptive use among married/in union women is 22%; that of unmet need among married/in union women is 30% and the demand for modern contraceptive satisfied is 39% [7]. With just 2 years to 2020, there is the need to increase efforts to satisfy women’s need for contraception. It is therefore imperative to look at the magnitude of the individual determinants and their effects on unmet need for contraception after accounting for unobserved household and/or cluster variations. Unlike contraceptive prevalence which does not consider women’s ability to become pregnant and their wishes for children unmet need for family planning, takes these factors into consideration. We therefore concentrated on unmet need for family planning which gives the vital information about women’s need for family planning.

## Methods

### Study design and data source

This study used a secondary data from the 2014 Ghana Demographic and Health Survey for the analysis. The 2014 Ghana Demographic and Health Survey (GDHS) is a nationally representative household survey that collects very wide range of population, health and other important indicators covering all the ten regions of Ghana. Participants in the survey were asked retrospective questions spanning 5 years prior to the survey.

### Sampling approach and study population

The 2014 GDHS followed a two-stage sample design and was intended to allow estimates of key indicators at the national level as well as for urban and rural areas and each of Ghana’s 10 administrative regions. The first stage involved selecting sample points (clusters) consisting of enumeration areas (EAs) delineated for the 2010 Ghana population and housing census (PHC). A total of 427 clusters were selected; 216 in urban areas and 211 in rural areas.

The second stage involved the systematic sampling of households. A household listing was undertaken in all the selected EAs in January–March 2014. The households included in the survey were randomly selected from the list. About 30 households were selected from each cluster to constitute the total sample size of 12,831 households. Because of the approximately equal sample sizes in each region, the sample is not self-weighting at the national level, and weighting factors have been added to the data file so that the results will be proportional at the national level [5]. In this current study, a total of 6503 (married/union) out of the 10,357 reproductive age women data were analysed in the 2014 GDHS.

### Outcome variable, inclusion and exclusion criteria

The outcome variable of interest is unmet need for family planning. Unmet need for family planning was categorized into three; unmet need for spacing, unmet need for limiting and no unmet need. The categorization also conformed to the recently revised version of unmet need for family planning applied in DHS [10]. The number of participants who had their classification regarding unmet need for spacing, limiting and no unmet need after data manipulation and with only complete case analysis (respondents with no missing information) were 1708(26.26%), 2918(44.87%) and 1877(28.67%) respectively.

The inclusion criteria involved women in their reproductive ages, that is, 15–49 years and were either currently married or in a union. We included only married/ in union women with the reasonable assumption that they are exposed to regular sexual intercourse.

The exclusion criteria were married/in union women who had incomplete information (missing data).

### Statistical analysis

The current analysis used both descriptive and inferential methods. Descriptive statistics used included frequencies and percentages. Both bivariate and multivariable techniques were used to assess statistical associations between the outcome variable and the predictors. The bivariate technique was applied to obtain predictors that had a statistically significant relationship with the outcome of interest (unmet need for family planning). In this approach, factors that were statistically significantly associated with the outcome were obtained via a simple multinomial mixed effects logistic regression model as well as chi-squared test of independence with the help of their confidence intervals (CI) and *p*-values. *P*-value less than or equal to 0.10 was used to retain and include variables in the multivariable analysis to obtain the risk ratios as a measure of association.

Further analysis were carried out with four selected variables (religion, region, education and age) to obtain predictive probabilities which enabled us observe the association between these predictor variables and the outcome. These were randomly picked for the purpose exploration. Although the simple multinomial mixed effects logistic regression model is complex, we used it because of the need to adjust and obtain parameter estimates through a fixed effects (multivariable) model, outcome variable categorized into three levels (referred to as multinomial), nesting nature of the GDHS data (multilevel) and the need to account for the cluster effects (via a random effects approach) which is not included in the data set.

The Ghana DHS 2014, is structured in such a way that women were nested within households and households were further nested within clusters. Due to the hierarchical nature of this survey, it is very important that a multilevel regression model be used in order to obtain a more accurate and reliable estimates of the model parameters. This modelling approach ensures that between household and cluster variations are properly accounted for in order to avoid parameter over-estimation. In accounting for these variations, enumeration areas referred to as clusters were considered as a level-2 variable while that of respondents or individual-level variables were assigned level-1. This statistical approach was implemented in STATA via a Generalized Structural Equation Modelling (with the logit link function and robust variance estimator for the standard error) approach in the STATA (Stata Statistical Software: Release 14. College Station, TX: StataCorp LP) software. We used Stata default number of iterations with convergence tolerances log likelihood of 1e^ (− 7). Four different models via nesting were specified and the final interpretation of coefficients were based on the best model among them. The best model was arrived at through the use of the log-likelihood ratio test and the Akaike information criteria. The predictive probabilities that were calculated and presented graphically were obtained using the robust approach to estimate the standard errors with vce (unconditional) option for the margins.

### Model building with potential risk factors

The specifications of the models were based on variables that showed significant associations at the bivariate analysis with the Pearson’s chi-squared test statistic. Groupings of these variables were done according to socio-demographic, socio-economic, socio-cultural and psychosocial and other factors. Model-1 constituted our first model containing socio-demographic variables (region, age category and place of residence). Model-2 was formulated using Model-1 in addition to socio-economic factors (respondent’s educational level, wealth index of respondent’s household, respondent’s occupation and partner’s occupation). Model-3 involved Model-2 and socio-cultural factors (respondent’s religious beliefs and ethnicity). Model-3 was nested in Model-4 in addition to psychosocial and other factors (infrequent sex, partner’s opposition to contraceptive use, and fear of side effect). All these models were implemented via the multilevel modelling approach.

## Results

**Fig. 1:**
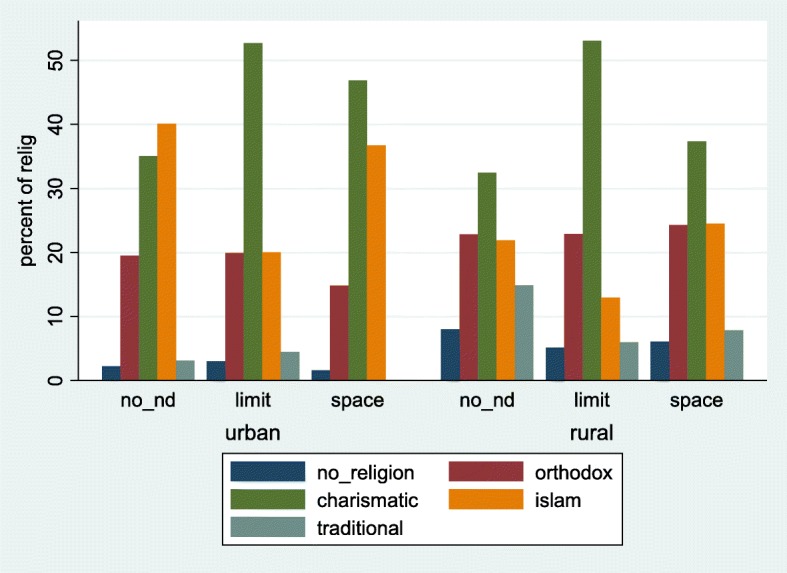
Distribution of unmet need for family planning among women (married or union) by religion and stratified by place of residence. Ghana Demographic and Health Survey, 2014. Legend: no_nd: no unmet need; limit: unmet need for limiting; space: unmet need for spacing

**Fig. 2:**
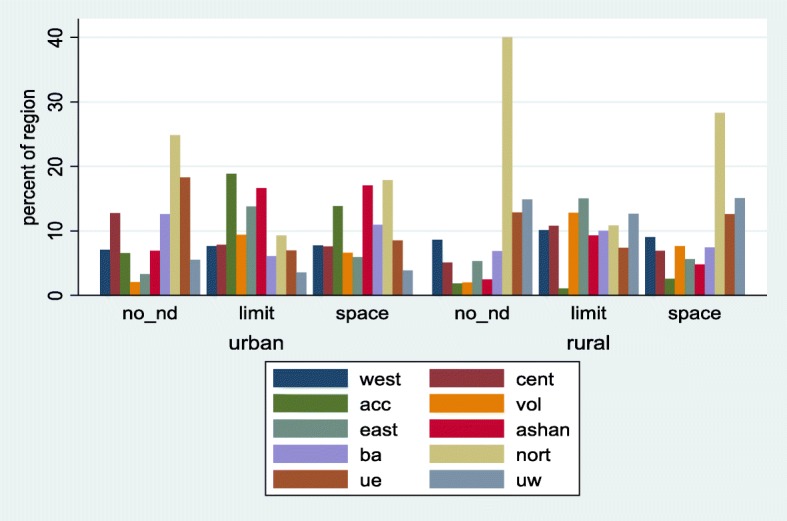
Distribution of unmet need for family planning among women (married or union) by region and stratified by place of residence. Ghana Demographic and Health Survey, 2014. Legend: no_nd: no unmet need; limit: unmet need for limiting; space: unmet need for spacing

**Fig. 3:**
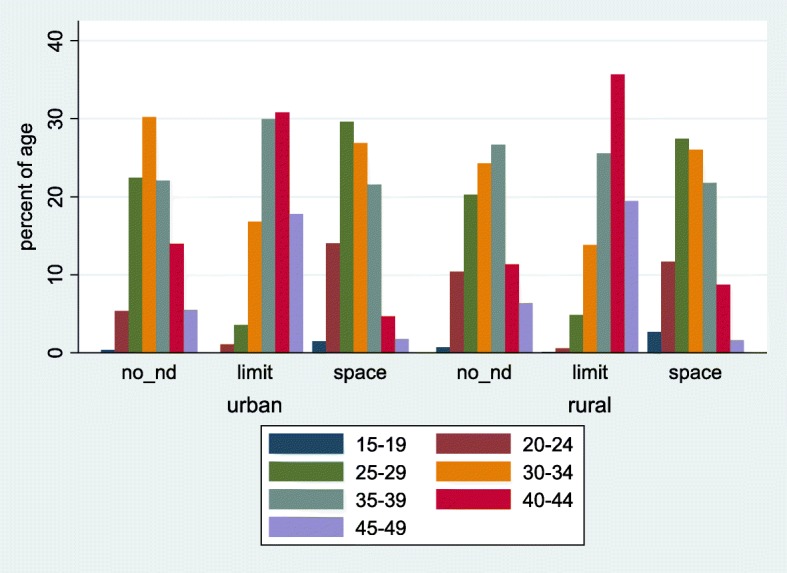
Distribution of unmet need for family planning among women (married or union) by age and stratified by place of residence. Ghana Demographic and Health Survey, 2014. Legend: no_nd: no unmet need; limit: unmet need for limiting; space: unmet need for spacing

**Fig. 4:**
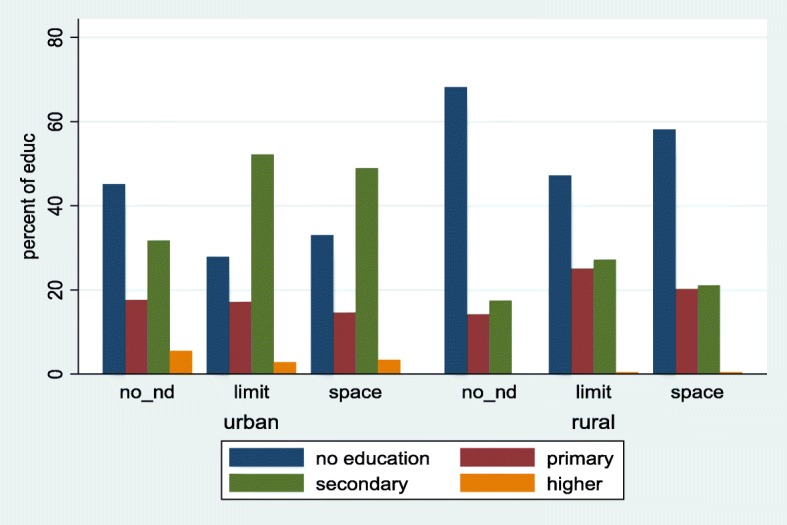
Distribution of unmet need for family planning among women (married or union) by education and stratified by place of residence. Ghana Demographic and Health Survey, 2014. Legend: no_nd: no unmet need; limit: unmet need for limiting; space: unmet need for spacing

A total of 6503 married/union women met the inclusion criteria for this study. The mean (standard deviation (SD) age in years of the women was 35.27(7.1). Out of the 6503, 1877(28.9%) had no unmet need for family planning, 2918(44.9%) had unmet need for limiting while 1708(26.3%) had unmet need for spacing. The mean (SD) age in years of the respondents with no unmet need was 33.13(6.7) while that for those with unmet need for limiting and spacing were 39.19(5.7) and 30.94(6.1) respectively. Figures 1, 2, 3, 4, depict percentages of the four main variables (religion, region, age category and educational level) that were of primary interest in this study and grouped according to the outcome variable (unmet need for family planning) and further grouped according to place of residence (urban and rural). Majority (40.1%) of the respondents who had no unmet need were of the Islamic religion for urban setting. Those with unmet need for limiting and spacing were of the Charismatic religious belief for both the urban and rural residence. Respondents without any religious background had the least type of unmet need across both residential types.

With respect to regional distribution, majority of those with no unmet need for family planning were from the Northern region of Ghana for both urban (24.9%) and rural (40.1%) areas. Similarly, women in the Northern region had the highest unmet need for spacing for both urban (17.9%) and rural (28.3%) areas. Concerning unmet need for limiting, the Greater Accra region reported the highest (18.8%) for the rural areas while the Eastern region had the highest (15.0%) for the urban areas.

There was a high cluster effect at the multivariable analyses level. There were 8.88 and 3.95 for limiting and spacing respectively, Table 1. The adjusted relative risk ratio results presented in Table 1, constitute one out of the four Models specified in the model building subsection, though it contains all the variables in the other sub-models. The final Model was arrived at after calculating the goodness of fit of all the Models using the likelihood ratio test statistic and the Akaike information criteria, as presented in Table S1 (Additional file 1). The best model was selected on the basis that it had the lowest value of the Akaike information criteria (AIC). As stipulated in (Additional file 1: Table S1, the more the significant variables were added to a Model, the better its fit. The Akaike information criteria was 8783.67 for Model-4 with its closest value being 8936.00 for Model-3, indicating that Model-4 is a better fit compared to Model-3, The difference between the two Models was 129.33. The likelihood ratio test for Model 4 compared to Model-3 was 164.33 with a *p*-value < 0.001, reinforcing the point that Model-4 is a better fit Model. Model-4 was therefore used for the final analysis. The calculated unobserved effect for the best fit Model (Model 4) was 8.88 implying a standard deviation of 2.98 for limiting. That for spacing was 3.95 implying a standard deviation of 1.99. The covariance between limiting and spacing was 2.73, an indication of a weak correlation (0.46) between them. Thus a 1-standard deviation of the random effects amounts to an exp. (2.98) = 19.69 and exp.(1.99) = 7.32 significant change in the relative risk ratio for limiting and spacing. Due to the type of model specified for these analyses, results are reported as relative risk ratios instead of odds ratios as expected if binary logistic regression is used for the analysis.

**Table 1:** Crude and adjusted risk ratios for unmet need for limiting / unmet need for spacing versus no unmet need for family planning versus among women (married or union)

Variables (chi-square & *p*-value)	Levels	Number (%)	Crude Risk Ratio (Limiting)	Crude Risk Ratio (Spacing)	Adjusted Risk Ratio (Limiting)	Adjusted Risk Ratio (Spacing)	Overall *P*-value
Region (Χ2 = 836.36, *p* < 0.001)							< 0.001 (limit) =0.003 (space)
	Volta	523(7.80)	Ref		Ref		
	Western	654 (8.71)	0.05 (0.01,0.20)	0.14 (0.05,0.44)	0.02 (0.00,0.09)	0.38 (0.10,1.42)	
	Central	584 (8.71)	0.10 (0.02,0.37)	0.13 (0.04,0.41)	0.03 (0.01,0.16)	0.24 (0.06,0.92)	
	Accra	886 (13.23)	0.18 (0.05,0.71)	0.38 (0.12,1.22)	0.10 (0.02,0.59)	0.63 (0.16,2.44)	
	Eastern	650 (9.70)	0.38 (0.10,1.43)	0.22 (0.07,0.71)	0.12 (0.02,0.60)	0.25 (0.07,0.94)	
	Ashanti	1207 (18.02)	0.31 (0.08,1.17)	0.47 (0.15,1.48)	0.10 (0.02,0.54)	0.80 (0.21,3.12)	
	Brong Ahafo	446 (6.65)	0.04 (0.01,0.14)	0.14 (0.04,0.41)	0.01 (0.00,0.05)	0.19 (0.05,0.69)	
	Northern	1218 (18.18)	0.01 (0.00,0.04)	0.07 (0.02,0.22)	0.00 (0.00,0.01)	0.18 (0.05,0.68)	
	Upper East	307 (4.59)	0.02 (0.00,0.56)	0.08 (0.03,0.25)	0.00 (0.00,0.01)	0.15 (0.04,0.60)	
	Upper West	225 (3.35)	0.04 (0.01,0.14)	0.12 (0.04,0.40)	0.01 (0.00,0.03)	0.20 (0.05,0.84)	
Age (Χ2 = 1.7e+ 03, *p* < 0.001)							< 0.001 (limit) < 0.001 (space)
	15–19	56 (0.84)	Ref		Ref		
	20–24	448 (6.69)	1.89 (0.29,12.18)	0.35 (0.15,0.83)	1.73 (0.22,13.36)	0.29 (0.12,0.71)	
	25–29	1032 (15.41)	7.82 (1.29,47.33)	0.35 (0.15,0.82)	9.13 (1.25,66.50)	0.34 (0.14,0.82)	
	30–34	1444 (21.55)	47.14 (7.75,286.66)	0.25 (0.11,0.59)	52.40 (7.17,382.89)	0.23 (0.09,0.54)	
	35–39	1719 (25.67)	105.85 (17.43,642.98)	0.21 (0.09,0.49)	133.08 (18.12,977.18)	0.17 (0.68,0.40)	
	40–44	1383 (20.65)	792.02 (128.88,4867.48)	0.16 (0.07,0.39)	> 1000	0.12 (0.05,0.30)	
	45–49	616 (9.19)	> 1000	0.07 (0.03,0.18)	> 1000	0.08 (0.03,0.20)	
Place of residence (Χ^2^ = 14.89, *p* = 0.001)							=0.060 (limit) =0.364 (space)
	Urban	2687 (40.12)	Ref				
	Rural	4012 (59.88)	0.72 (0.39,1.31)	0.63 (0.39,1.00)	0.76 (0.36,1.63)	0.74 (0.42,1.32)	
Educational level (Χ2 = 208.55, *p* < 0.001)							< 0.001 (limit) =0.354 (space)
	No education	2635 (39.33)	Ref				
	Primary	1339 (19.98)	0.82 (0.64,1.05)	1.31 (1.02,1.69)	2.37 (1.68,3.35)	1.04 (0.78,1.40)	
	Secondary	2574 (38.42)	0.76 (0.59,0.98)	1.58 (1.22,2.04)	2.08 (1.43,3.04)	1.34 (0.97,1.84)	
	Higher	152 (2.27)	0.260 (0.13,0.54)	0.75 (0.35,1.61)	0.58 (0.20,1.70)	0.53 (0.20,1.42)	
Wealth index (Χ2 = 265.18, *p* < 0.001)							< 0.001 (limit) =0.094 (space)
	Poorest	1992 (29.74)	Ref				
	Poorer	1541 (23.00)	0.86 (0.65,1.13)	1.90 (1.44,2.51)	0.50 (0.34,0.73)	1.90 (1.38,2.61)	
	Middle	1185 (17.68)	0.86 (0.59,1.25)	2.58 (1.79,3.73)	1.00 (0.59,1.68)	2.26 (1.45,3.52)	
	Richer	1038 (15.49)	0.94 (0.61,1.47)	2.56 (1.68,3.90)	0.70 (0.36,1.35)	1.79 (1.03,3.10)	
	Richest	943 (14.08)	0.26 (0.15,0.44)	1.75 (1.10,2.80)	0.16 (0.07,0.35)	1.32 (0.70,2.51)	
Respondent’s occupation (Χ2 = 160.39, *p* < 0.001)	No work	861 (12.87)	Ref				< 0.001 (limit) =0.264 (space)
	Professional	129 (1.94)	3.06 (1.45,6.43)	0.64 (0.29,1.41)	6.06 (2.05,17.92)	1.00 (0.36,2.75)	
	Sales	2386 (35.67)	4.21 (3.05,5.83)	1.00 (0.75,1.32)	2.25 (1.48,3.40)	1.06 (0.77,1.44)	
	Agriculture	2491 (31.26)	5.63 (4.10,7.74)	0.82 (0.63,1.07)	2.92 (1.93,4.41)	1.20 (0.89,1.62)	
	Services	65 (0.66)	8.86 (3.17,24.77)	0.90 (0.33,2.46)	16.92 (4.78,59.97)	0.60 (0.19,1.88)	
	Manual	754 (11.28)	4.82 (3.28,7.08)	0.97 (0.69,1.38)	3.98 (2.43,6.53)	1.07 (0.73,1.57)	
Partner’s occupation (Χ2 = 26.84, *p* = 0.001)							=0.003 (limit) < 0.001 (space)
	Professional	519 (7.82)	Ref				
	Sales	580 (8.75	0.91 (0.57,1.46)	1.77 (1.09,2.85)	0.79 (0.42,1.49)	1.85 (1.09,3.14)	
	Agriculture	3359 (50.65)	1.38 (0.95,2.02)	1.08 (0.73,1.59)	0.68 (0.39,1.18)	1.47 (0.93,2.33)	
	Manual	2047 (30.86)	0.34 (0.17,0.70)	0.75 (0.37,1.54)	0.11 (0.04,0.27)	0.67 (0.29,1.53)	
	Services	127 (1.92)	0.53 (0.36,0.77)	0.69 (0.46,1.02)	0.47 (0.28,0.79)	0.60 (0.38,0.94)	
Religion (Χ2 = 300.89, *p* < 0.001)							=0.374 (limit) =0.912 (space)
	Traditional	364 (5.44)	Ref				
	No religion	288 (4.30)	0.90 (0.57,1.41)	1.42 (0.91,2.22)	2.58 (1.40,4.73)	1.22 (0.75,1.98)	
	Orthodox	1352 (20.19)	0.48 (0.32,0.70)	1.41 (0.97,2.05)	1.22 (0.72,2.07)	1.51 (1.01,2.27)	
	Charismatic	3300 (49.26)	0.88 (0.61,1.28)	1.76 (1.23,2.53)	2.83 (1.71,4.67)	1.66 (1.12,2.44)	
	Islam	1394 (20.81)	0.80 (0.50,1.26)	2.57 (1.65,3.98)	1.82 (0.96,3.44)	2.29 (1.39,3.76)	
Ethnicity (Χ2 = 717.90, *p* < 0.001)							=0.001 (limit) =0.002 (space)
	Akan	2741 (40.92)	Ref				
	Ga/Dangme	449 (6.70)	2.58 (1.29,5.13)	2.62 (1.27,5.39)	2.10 (0.87,5.06)	3.92 (1.73,8.90)	
	Ewe	852 (12.71)	1.48 (0.90,2.42)	3.09 (1.84,5.19)	0.54 (0.29,1.00)	3.47 (1.87,6.43)	
	Guan	124 (1.85)	0.10 (0.04,0.30)	2.47 (1.12,5.45)	0.23 (0.07,0.77)	3.43 (1.38,8.54)	
	Mole - dagbani	1371 (20.46)	0.41 (0.28,0.61)	1.05 (0.71,1.54)	0.73 (0.39,1.36)	1.61 (0.94,2.76)	
	Grusi	236 (3.52)	1.13 (0.61,2.10)	3.32 (1.82,6.05)	3.11 (1.28,7.54)	5.37 (2.55,11.34)	
	Gurma	700 (10.45)	0.09 (0.04,0.20)	0.81 (0.42,1.58)	0.27 (0.09,0.77)	1.07 (0.49,2.35)	
	Mande	71 (1.06)	0.33 (0.14,0.80)	1.42 (0.67,3.03)	0.09 (0.03,0.29)	2.07 (0.86,4.99)	
Infrequent sex (Χ2 = 35.38, *p* < 0.001)							< 0.001 (limit) < 0.001 (space)
	No	5983 (89.31)	ref				
	Yes	716 (10.69)	2.37 (0.60,1.13)	1.93 (1.48,2.51)	4.63 (3.26,6.57)	2.37 (1.77,3.16)	
Partner’s opposition (Χ2 = 18.79, *p* < 0.001)							=0.339 (limit) =0.117 (space)
	No	6279 (93.73)	Ref				
	Yes	420 (6.27)	0.79 (0.56,1.10)	0.70 (0.51,0.98)	0.77 (0.49,1.20)	0.77 (0.53,1.10)	
Fear of side effect (Χ2 = 359.93, *p* < 0.001)							< 0.001 (limit) < 0.001 (space)
	No	4046 (60.40)	ref				
	Yes	2653 (39.60)	2.96 (2.43,3.61)	2.44 (1.99,2.99)	2.94 (2.28,3.80)	2.58 (2.05,3.24)	
Variations Covariance (limiting & spacing)					8.88	3.95	2.731

Results from a multinomial mixed effect logistic regression model (Model 4 see Additional file 1: Table S1). Ghana Demographic and Health Survey, 2014

### Demographic determinants of unmet need for family planning

Table 1, shows both the bivariate and multivariable multinomial mixed effects logistic regression analyses results. Under the socio-demographic grouping approach with the adjusted relative risk ratio, there was a reduced risk of unmet need for limiting against no unmet need. A reduced risk of 99.9% (RR of 0.01 (95% CI, 0.00, 0.03, *p*-value < 0.001) was observed for people from the Upper West region compared to that of the Volta region. Also, a reduced risk of 88.8% (RR of 0.12 (95% CI, 0.22, 0.60, *p*-value = 0.01) for respondents from the Eastern region was observed against respondents from the Volta region. Similar observations for unmet need for spacing were made except that the highest relative risk among the ten regions was the Ashanti and Eastern regions for limiting as compared to no unmet need. Though the relative risk for the Upper West region was 0.20(95% CI, 0.05, 0.84, *p*-value = 0.028) and that of the Eastern region and Ashanti regions were 0.25 (95% CI, 0.07, 0.94, *p*-value = 0.040) and 0.80 (95% CI, 0.21, 3.12, *p*-value = 0.750) respectively, only Upper West was significant. This implies that the risk for unmet need for women from the Volta region in relation to those from the Upper West and Eastern regions for limiting were 205 and 9 times higher. That of spacing were 5 and 4 times higher. In terms of the age category, those within 15–19 were used as the reference group. The observations made were that a change in age from lower to higher corresponds to an increase risk of an unmet need for limiting compared to no unmet need. For instance, the risk of women aged 20–24 years had a risk ratio of 1.73 (95% CI, 0.22, 13.34, *p*-value = 0.600). The risk ratio for age group 35–39 years was 133 (95% CI, 18.12, 977.18, *p*-value < 0.001). The opposite was the case for all the year groups compared to the 15–19 years respondents for spacing. The risk of respondents aged 20–24 compared to 15–19 when evaluated under unmet need for spacing gave an RR of 0.29 (95% CI, 0.12, 0.71, *p*-value < 0.007) and that of 35–39 had an RR of 0.17 (95% CI, 0.07, 0.40, *p*-value < 0.001) times the risk for no unmet need. This shows that respondents within 15–19 years group were 3 and 6 times more likely to develop the need for spacing as against the 20–24 and 35–39 age groups. All the other age groupings were similarly related.

**Fig. 5:**
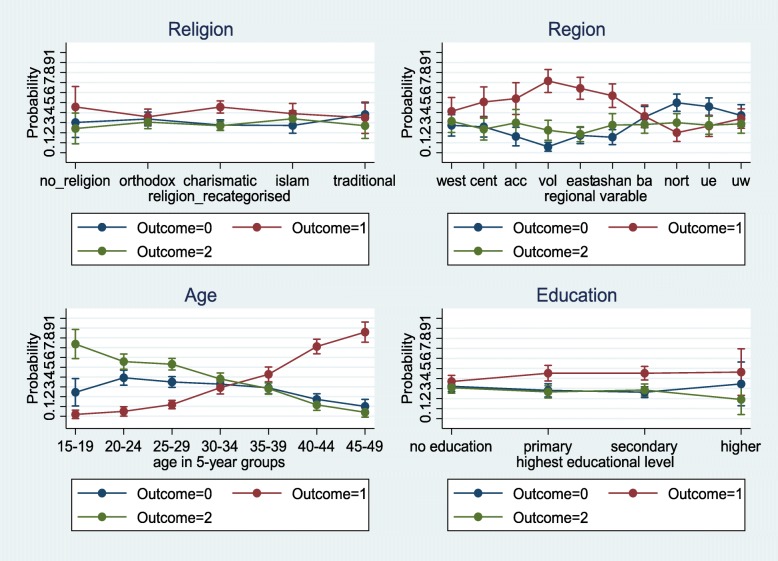
Adjusted probability of unmet need for family planning (no unmet need: outcome = 0); unmet need for limiting (outcome = 1); unmet need for spacing (outcome = 2)) among women (married or union). Results from a multinomial mixed effect logistic regression model. Ghana Demographic and Health Survey, 2014

Figure 5, contains the adjusted predictive probabilities of the types of unmet need for family planning according to regions and age categories of respondents. The marginal predictive probabilities for the unmet need for limiting is highest among respondents from the Volta region (0.80) followed by the Eastern region (0.66) with the smallest being the three regions of the Northern part of the country. For the age category, the marginal probabilities for limiting increased upwardly with a higher age. The predictive probability for wanting to limit was 0.86 for the 45–49 year group and as low as 0.02 among the 15–19 year group.

### Socio-economic determinants of unmet need for family planning

Four socio-economic factors were identified to be statistically significantly associated with unmet need for family planning. These were respondent’s educational level, wealth index of household, respondent’s occupation and partner’s occupation. With respect to wealth index, only the richest and poorer respondents showed a significant difference. The middle and the richer were insignificant statistically when compared to the poorest with regards to unmet need for limiting. Respondents who were poorest have 2 times (95% CI, 1.36, 2.97, *p*-value < 0.001) the risk of having an unmet need for limiting compared to the poorer respondents. Under spacing, the richest had approximately 32% more risk. The poorer had 89%more risk than the poorest respondents. Educational level did not demonstrate any statistical significant difference for spacing. For limiting, respondents with primary and secondary education had about 2 times the risk with a *p*-value < 0.001. With regards to respondent’s occupation, those in the services and professionals had an RR of 16.92 times (95% CI, 4.78, 59.97, *p*-value < 0.001) and 6.06 times (95% CI, 2.05, 17.92, *p*-value = 0.001) risk of having unmet need for limiting compared with those without any work. The marginal probabilities for educational levels, presented in Fig. 5, shows that, the predictive probability for respondents classified under primary was the highest (0.47), followed by secondary (0.44) for limiting.

### Socio-cultural determinants of unmet need for family planning

Respondents, religion and ethnicity were the only socio-cultural variables statistically significantly associated with unmet need for family planning. Religion was re-categorized into no religion, Orthodox, Charismatic, Islamic and Traditional for further analysis. The results showed that respondents without any religious affiliation had more than twice (with a *p*-value = 0.002) and 22% (with a *p*-value = 0.430) the risk of experiencing unmet need for limiting and spacing respectively compared to those with in traditional religion. Unmet need for family planning for the Charismatic group was approximately 3 with a *p*-value < 0.001 and 1.65 with a *p*-value < 0.011 times the risk for limiting and spacing than it was for traditional religion. From Fig. 2, a higher predictive probability (0.46) was observed for respondents without any religious affiliation and those with the Charismatic faith (0.46) for limiting.

### Psychosocial and other determinants of unmet need for family planning

All the variables identified under this category were insignificant except those who reported infrequent sex, partner’s opposition to use of contraceptives and respondents fear of side effects. Respondents who fear contraceptive side effects were 3 times (95% CI, 2.28, 3.80 *p*-value < 0.001) at risk of having unmet need for limiting and 2.58 times (95% CI, 2.05, 3.24, *p*-value < 0.001) more likely to experience an unmet need for spacing when compared to respondents who do not fear contraceptive side effects. Respondents who had infrequent sex were 4.6 times more likely to want to limit and 2.4 times more likely to space their children than those who had frequent sex.

## Discussion

Making use of the data for women in the reproductive age in the 2014 GDHS, this study used the most appropriate statistical model that has the power to control for unobserved effect estimates in the data set to determine the significant factors associated with unmet need for family planning in Ghana. This knowledge is important for policy makers and service providers to enable them put pragmatic measures in place to satisfy the unmet need for family planning.

Our study showed that a number of socio-demographic (age, religion and administrative region of residence), socio-economic (wealth index, respondents educational level, respondent’s and partner’s occupation), cultural (religion and ethnicity) as well as fear of contraceptive side effects, infrequent sex and opposition from partners were are all significant determinants of both unmet need for limiting and spacing.

Our analysis showed an upward trend of limiting for higher age groups. As women’s age changed from 15 to 19 group to 20–24 group, the likelihood of having an unmet need for FP only doubled but when 15–19 group was compared to 45–49 group, unmet need increased more than a thousand fold. For spacing, the likelihood of an unmet need decreased with an increasing age group. A similar conclusion was arrived at in a study by Wafula et al., in Kenya [11]. These findings are likely to be due to the fact that young women had not attained their desired family size and therefore their need is to space their children. On the other hand, older women might have attained their desired family size and would therefore not like to have any more children.

Religion was also observed to be a factor leading to having a higher unmet need for family planning as was found in other studies in India and Ethiopia [12–14]. Compared to those who professed traditional religion, women with no religious affiliation and those with the charismatic faith were twice more likely to have an unmet need for limiting. Women practicing Islamic religion were less likely to space birth compared with those practicing traditional religion. The different religious beliefs have varied perceptions and self-beliefs that could impact either negatively or positively in contraceptive use [11]. Members of Islamic religion and some Orthodox religions such as Catholics exhibit a strong opposition to contraceptive use. Overall, women who belonged to other religious beliefs other than traditional religion appeared to have a higher unmet need for limiting and spacing as compared to respondents who belonged to the traditional religion [11].

With regards to education, this study revealed a higher unmet need for both limiting and spacing among respondents who had completed either primary or secondary education compared to those without any formal education; similar conclusions were drawn from other studies [15, 16]. A non-significant effect between higher and no educated respondents were observed and this is contrary to findings from Kenya [11]. They observed a higher unmet need for women with low educational background. The high unmet need for family planning in educated Ghanaian women may explain why induced abortion tends to be higher in them as compared to women with no education [17, 18]. It has been suggested that induced abortion may be an integral factor in the control of fertility among educated Ghanaian women [17]. It is also possible that highly educated women may have knowledge about potential contraceptives side effects which may translate into low use among this demographic group. It was further observed that place of residence was a statistically insignificant contributor to unmet need for family planning, though rural residents were less likely to have an unmet need. This finding is again contrary to findings of Genet et al., in Ethiopia [19], which stipulated that rural respondents were twice more likely to have unmet need. There are a number of possibilities that could have influenced our findings. Family planning services have also been an integral part of health services provided in rural areas in Ghana and this high level of awareness created in these areas may have had positive impact on FP.

Our study has also shown that, the fear of side effects, infrequent sexual intercourse and opposition from partners are all significant factors contributing to the high unmet need for family planning in Ghana. Similarly, demographic and health surveys from 52 countries spanning the period from 2005 to 2014 have shown that about 7 out of 10 married women with unmet need for family planning cite either fear of side effects or health risks, infrequent or no sex and opposition to contraception (either by they themselves or from significant others) as their reason for not using modern contraception [20]. This is an indication that satisfying the needs of women with unmet need for family planning will get a big boost if these factors are tackled with the seriousness they deserve.

In many countries contraceptive prevalence have stalled and this has been attributed partly to the poor quality of counselling and hence the call for new approaches to counselling [21]. Good counselling should pay attention to dealing with misconceptions, how to prepare new clients to handle common side effects and also how continuing clients can cope with side effects [22].

A recent study from five urban family planning centres in Ghana revealed that even though over two thirds of women adopting a family planning methods were counselled to expect side effects, over a third of these same women were not counselled on common side effects of their chosen methods [23]. In the same study, about 7 out of 10 family planning acceptors chose methods whose side effects they had stated earlier will cause them to stop the said method. This shows that much importance was not attached to side effects of clients before they were given their chosen methods. In addition, women wary of side effects could also be educated on natural FP methods which they may not be familiar with. Studies have shown that mobile application for contraception based on a woman’s natural cycle is effective in preventing pregnancies [24]. Such tools on fertility-awareness may be the solution for women for whom side effects are positive predictors of unmet needs on limiting and spacing [23, 24]. Quality of family planning services which includes, good counselling is associated with clients selecting family planning methods that best suits their individual needs. This will enable them navigate through side effects effectively and to continue to use their choice of methods [25].

Respondents who had infrequent sexual intercourse were about four and two times more likely to have an unmet need for limiting and spacing respectively. It is possible that women who had infrequent sexual intercourse may not want to be to be using a method continuously when they do not know when they next will have sexual intercourse. They are however at risk of unintended pregnancies and need to be abreast with emergency contraception and barrier methods in order to avoid unintended pregnancies.

For those whose partners oppose their contraceptive use, there will be the need to get them involved in order for them to appreciate the benefits of family planning. Some men have the wrong impression that their spouses may become promiscuous once they are using contraceptives [26].

While acknowledging that factors such as level of education, wealth index and religion will require multi-sectoral approach to handle, dealing with side effects, getting women with infrequent sex to use emergency contraception or barrier methods, and educating partners on the benefits of family planning lies mostly in the domain of service providers. There is the need to start dealing with the high unmet need for family planning by tackling these three factors first.

### Strengths and limitations of the study

This study derives its strengths from the fact that, the use of a nationally representative sample allows for the generalizability of study findings to the whole country. In addition, demographic and health surveys are well planned and executed surveys and therefore the data is usually of high quality. Furthermore, the number of observations with complete dataset that met the inclusion criteria was large. The use of the multilevel mixed effects logistic model addresses the issues of cluster variations by appropriately accounting for those unobserved effects that are not usually measured by the dataset. The coefficient estimates obtained in this study are more accurate and generalizable due to our modelling approach.

This study also had some limitations. To begin with, the data was obtained through a cross-sectional study and so causations could not be established. Secondly, there were delays in model convergence due to the complex nature of the model proposed and applied to the dataset but that did not have an effect on the parameters estimates. The survey obtained retrospective information which was self-reported from participants spanning a 5-year period prior to the survey and so the likelihood of recall bias was high. Recall bias has some consequences on coefficient estimates and overall significant testing and so interpretations/use of the results should be done cautiously.

## Conclusions

This study reveals that socio-demographic factors such as respondents region, age and not place of residence contribute to predicting unmet need for family planning. Also, socio-economic (partner’s occupation) and cultural (religion and ethnicity) as well as side effects are all significant determinants of both unmet need for limiting and spacing. Variables such as, educational level, wealth index and respondents occupation overall were significant in predicting only unmet need for limiting but insignificant for predicting unmet need for spacing. The fear of side effect on the use of contraceptives as well as infrequent sex among respondents are both high predictors of unmet need for family planning. Overall, fear of side effect, infrequent sex, age, ethnicity, partner’s education and region were the most highly significant predictors of both limiting and spacing. The Ministry of Health need to work more closely with the Ghana Health Service to train its service providers to ensure that prospective family planning acceptors are counselled adequately on common side effects of their methods of choice and to also address misconceptions. All stakeholders in family planning must do their best to extol the virtues of family planning to men and help involve them in family planning.

## Additional file

Additional file 1: **Table S1.** Model building strategy: unmet need for family planning among women (married or union), Ghana Demographic and Health Survey, 2014. (DOCX 27 kb)

## Abbreviations

CI: Confidence Interval
exp.: Exponential
FP: Family Planning
GDHS: Ghana Demographic Health Survey
IUD: Intrauterine Device
RR: Risk Ratio
