# Supplementary material for: Factors influencing unmet need for family planning among Ghanaian married/union women: a multinomial mixed effects logistic regression modelling approach
Source: Arch Public Health. 2019 Mar 12;77:11. doi: 10.1186/s13690-019-0340-6 (PMC6413448; doi:10.1186/s13690-019-0340-6)
Supplement: Supplementary file 1 — Table S1. Model building strategy: unmet need for family planning among women (married or union), Ghana Demographic and Health Survey, 2014. (DOCX 27 kb) [file 13690_2019_340_MOESM1_ESM.docx]

**Model Building**

**Table S1**, Model building strategy: unmet need for family planning among women (married or union), Ghana Demographic and Health Survey, 2014

| **Testing** | **Model-1** | **Model-2** | **Model-3** | **Model-4** |
| --- | --- | --- | --- | --- |
|  | Regions, respondents’ age category and respondents’ place of residence | Model-1 + respondents’ educational level, occupation, wealth index, and partners’ occupation | Model-2 + respondents’ religion and ethnicity | Model-3 + infrequent sex, partners’ opposition and fear of side effect |
| **Model fit** |  |  |  |  |
| Likelihood ratio test | - | 291.72  (p-value<0.001) | 113.43  (p-value<0.001) | 164.33  (p-value<0.001) |
| Akaike information criteria | 9233.15 | 9021.43 | 8935.996 | 8783.67 |
| Determination of the better fit Model |  | An indication of Model-2 being a better fit over Model-1 | Model-3 is a better fit over Model-2 | An indication of overall better fit Model over the previous ones |
